# Supplementary material for: Real-World Outcomes and Treatments Patterns Prior and after the Introduction of First-Line Immunotherapy for the Treatment of Metastatic Non-Small Cell Lung Cancer
Source: Cancers (Basel). 2022 Sep 15;14(18):4481. doi: 10.3390/cancers14184481 (PMC9497168; doi:10.3390/cancers14184481)
Supplement: Supplementary file 1 [file cancers-14-04481-s001.zip › cancers-1878015-supplementary.pdf]

Table S1. Second- and third-line treatments administrated by Pre 1L and Post 1L IO cohort.

**Therapies**	**Pre 1L IO**		**Post 1L IO**		
	**2L**	**3L**	**2L PD-L1 TPS ≥ 1%**	**2L PD-L1 TPS < 1%**	**3L**
	***n* = 151 (%)**	***n* = 65 (%)**	***n* = 61 (%)**	***n* = 71 (%)**	***n* = 45 (%)**
**Multi-agents chemotherapy**	**21 (13.9)**	**12 (18.6)**	**13 (21.3)**	**5 (7.0)**	**6 (13.3)**
**Single agent chemotherapy**	**45 (29.8)**	**22 (33.8)**	**6 (9.8)**	**15 (21.2)**	**28 (62.2)**
Vinorelbine	17 (11.3)	11 (16.9)	5 (8.2)	5 (7.0)	8 (17.8)
Docetaxel	19 (12.5)	7 (10.8)	1 (1.6)	7 (9.9)	11 (24.4)
Other agents	9 (6.0)	4 (6.1)	0 (0.0)	3 (4.2)	9 (20.0)
**Targeted therapy**	**32 (21.2)**	**9 (13.8)**	**0 (0.0)**	**2 (2.8)**	**1 (2.2)**
Erlotinib	28 (18.6)	4 (6.2)	0 (0.0)	0 (0.0)	0 (0.0)
Other agents	4 (2.6)	5 (7.6)	0 (0.0)	2 (2.8)	1 (2.2)
**PD-1/PD-L1 inhibitor single agent**	**3 (2.0)**	**3 (4.6)**	**38 (62.3)**	**47 (66.2)**	**7 (15.6)**
Nivolumab	0 (0.0)	0 (0.0)	12 (19.7)	22 (31.0)	4 (8.9)
Atezolizumab	1 (0.7)	3 (4.6)	14 (22.9)	25 (35.2)	3 (6.7)
Pembrolizumab	2 (1.3)	0 (0.0)	12 (19.7)	0 (0.0)	0 (0.0)
**PD-1/PD-L1 inhibitor + chemotherapy**	**0 (0.0)**	**0 (0.0)**	**0 (0.0)**	**0 (0.0)**	**0 (0.0)**
**Clinical trials**	**50 (33.1)**	**19 (29.2)**	**4 (6.6)**	**2 (2.8)**	**3 (6.7)**
ICIs agents	45 (29.8)	18 (27.7)	1(1.6)	0 (0.0)	2(4.5)
Other agents	5 (3.3)	1 (1.5)	3(5.0)	2 (2.8)	1(2.2)

The second line of Post-1L IO cohort was further grouped based on PD-L1(TPS ≥ 1%; TPS < 1%).

Table S2. Outcome data by cohort. rwPFS and real-world response were associated with first-line treatment.

	**Pre 1L IO**<br>***n* = 344 (%)**	**Post 1L IO**<br>***n* = 350 (%)**
***n* of evaluable patients for OS**	344	350
***n* of events considered for OS**	329	253
**Median OS, months (95%CI)**	6.2 (5.5-7.4)	8.9 (7.5-10.6)
**% OS rate at 6 months (95%CI)**	51.2 (45.8-56.3)	59.1 (53.8-64.1)
**% OS rate at 12 months (95%CI)**	28.2 (23.5-33.0)	42.5 (37.2-47.8)
**% OS rate at 18 months (95%CI)**	19.2 (15.2-23.5)	31.9 (26.7-37.3)
**% OS rate at 24 months (95%CI)**	13.9 (10.5-17.8)	22.7 (17.7-28.1)
***n* of patients evaluable for rwPFS**	329	336
***n* of progressive disease (%)**	328	291
**Median rwPFS, months (95%CI)**	3.7 (3.3-4.2)	4.7 (3.9-5.7)
**% rwPFS rate at 3 months (95%CI)**	58.7 (53.1-63.8)	63.9 (58.6-68.9)
**% rwPFS rate at 6 months (95%CI)**	29.5 (24.7-34.5)	42.9 (37.5-48.1)
**% rwPFS rate at 12 months (95%CI)**	9.1 (6.3-12.5)	23.9 (19.4-28.8)
**% rwPFS rate at 18 months (95%CI)**	3.9 (2.2-6.4)	13.1 (9.4-17.4)
**CR as best overall response**	1 (0.5)	13 (4.7)
**PR as best overall response**	51 (24.4)	83 (29.7)
**SD as best overall response**	62 (29.7)	77 (27.6)
**PD as best overall response**	95 (45.5)	106 (38.0)
**Not evaluable**	92	121
***n*. of evaluable pts for response or disease control**	240	271
**Number of rw ORR**	66	85
**Rate (95%CI)**	27.5 (21.9-33.6)	31.4 (25.9-37.2)
**Number of rwDCR**	134	160
**Rate (95%CI)**	55.8 (49.3-62.2)	59.0 (52.9-64.9)

Table S3.. Outcome data for subgroup of Post-1L IO patients, who received IO in first-line treatment.

	**Post 1L IO** ***n* = 71 (%)**
***n* of evaluable patients for OS**	71
***n* of events considered for OS**	37
**Median OS, months (95%CI)**	15.5 (9.8-23.5)
**% OS rate at 6 months (95%CI)**	73.2 (61.3-82.0)
**% OS rate at 12 months (95%CI)**	58.9 (46.2-69.6)
**% OS rate at 18 months (95%CI)**	45.7 (32.2-58.2)
**% OS rate at 24 months (95%CI)**	34.7 (20.5-49.3)
***n* of patients evaluable for rwPFS**	68
***n* of progressive disease (%)**	44
**Median rwPFS, months (95%CI)**	12.1 (6.8-15.1)
**% rwPFS rate at 3 months (95%CI)**	77.9 (66.1-86.1)
**% rwPFS rate at 6 months (95%CI)**	66.2 (53.6-76.1)
**% rwPFS rate at 12 months (95%CI)**	50.1 (37.5-61.5)
**% rwPFS rate at 18 months (95%CI)**	31.4 (9.6-43.9)
